# Complete plastid genome of *Dendrobium naungmungense* (Orchidaceae)

**DOI:** 10.1080/23802359.2019.1674736

**Published:** 2019-10-09

**Authors:** Min-Hua Wang, Liang Ma

**Affiliations:** College of Landscape Architecture, Fujian Agriculture and Forestry University, Fuzhou, PR China

**Keywords:** Dendrobiinae, *Dendrobium*, plastid genome, phylogeny

## Abstract

*Dendrobium* is one of the most important genera in Orchidaceae. In this study, we used the next-generation sequencing technology and assembled a complete plastid genome of a recently published new species of *Dendrobium*, *D. naungmungense*. The plastome was 151,883 bp in length, containing a large single-copy region (LSC) of 87,189 bp, and a small single-copy region (SSC) of 12,150 bp, and two inverted repeat regions (IR) of 26,272 bp. A total of 123 genes were predicted, including 38 tRNA genes, 8 rRNA genes, and 77 protein-coding genes. Phylogenetic analysis of 44 representative plastome of the genus *Dendrobium* and outgroup suggested *D. naungmungense* to be sister to *Dendrobium wardianum.*

*Dendrobium* Swartz (1799, p. 82) (Orchidaceae: Epidendroideae; Malaxideae) is one of the largest genera of Orchidaceae with highly medicinal and ornamental value, which contains approximately 1600 species (Wu et al. 2009; Chiang et al. 2012), and is mainly distributed in tropical and subtropical Asia, and northern and eastern Australia (Wood 2006; Zhu et al. 2009). *Dendrobium naungmungense* is a new species from Naungmung, Kachin State, North Myanmar discovered in 2018 (Liu et al. 2018). *Dendrobium naungmungense* is close to *D. vexabile* and *D. ciliatilabellum* in morphological characteristics, but it is different with the oblong epichile, three long-ciliate laminae, the margin crisped with hairs and the margin of column wing with significant denticulation (Liu et al. 2018). In this study, we get the plastid genome of *D. naungmungense* to clarify the feature of the plastid genome and whether the molecular phylogeny relationship is corresponding with the morphological characteristics.

Fresh Leaf sample of *D. naungmungense* was acquired from Jiele Reservoir (24°03′N, 97°53′E), Ruili City, Dehong Dai and Jingpo Autonomous Prefecture, Yunnan Province of China, and voucher specimen deposited at Herbarium of Fujian Agriculture and Forestry University (specimen code FJFC 20170642). The total genomic DNA was extracted from Fresh leaves tissue, with 400 bp randomly interrupted by the Covaris ultrasonic breaker for library construction. The constructed library was sequenced by Illumina Hiseq 4000 platform, approximately 20GB data generated. The clean reads were used to assemble the complete chloroplast genome by the GetOrganelle pipe-line (Jin et al. 2018) with the chloroplast genomes of *Dendrobium* as the reference sequences. Then assembled plastid genome annotation by Geneious 11.1.5 (Kearse et al. 2012) based on comparison with *Dendrobium wardianum* (LC192961).

The complete plastid genome of *D. naungmungense* (GenBank accession MN481284) is 151,883 base pairs (bp) in length, containing a large single-copy (LSC) region of 87,189 bp, a small single-copy (SSC) region of 12,150 bp, and two inverted repeat (IR) regions of 26,272 bp. The new sequence possesses total 123 genes, including 77 protein-coding genes, 8 rRNA genes, and 38 tRNA genes. The overall GC content of the chloroplast genome was 37.45%, while the corresponding values of the LSC, SSC, and IR regions are 34.97%, 29.47%, and 43.42%, respectively. To confirm the phylogenetic position of *D. naungmungense*, 44 representative species of *Dendrobiinae* and outgroup species were aligned using MAFFT version 7.307 (Katoh and Standley 2013), and phylogenetic tree constructed by RAxML (Stamatakis 2014) (Figure 1). The ML tree showed the genus *Dendrobium* is monophyletic, and *D. naungmungense* is sister to *D. wardianum* (GenBank accession LC192961), with strong support and different from the morphological.

**Figure 1.**
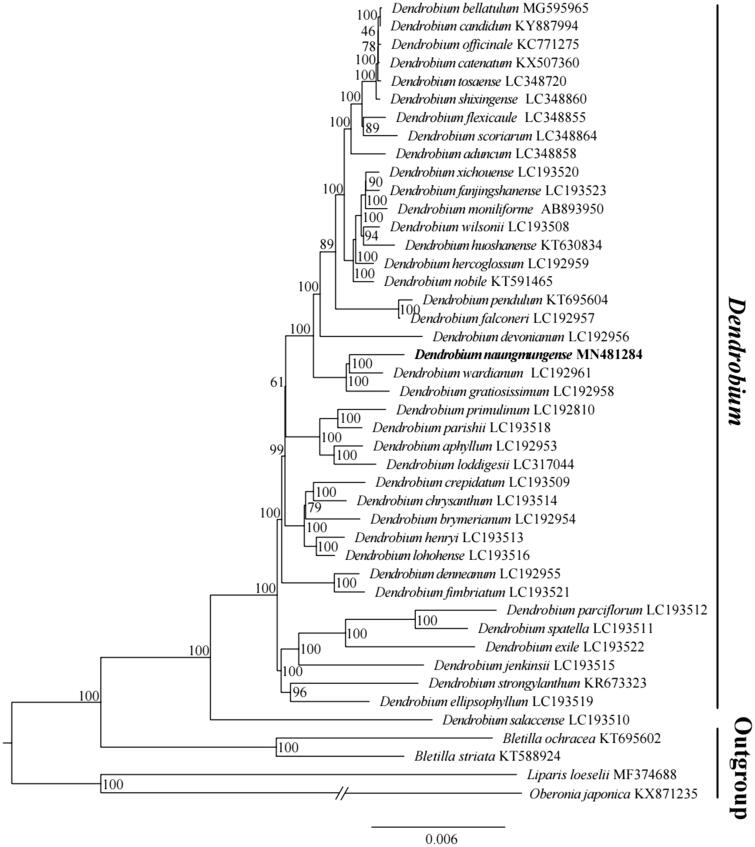
The maximum-likelihood (ML) tree based on the 44 representative plastid genomes of the *Dendrobium* and outgroup. The bootstrap value based on 1000 replicates is shown near each node.
